# Malaria in the returning older traveler

**DOI:** 10.1186/s40794-016-0018-9

**Published:** 2016-02-04

**Authors:** N Allen, C Bergin, SP Kennelly

**Affiliations:** 1 Department of Genitourinary medicine and Infectious Diseases, St. James’s Hospital, Dublin 8, Ireland; 2 Department of Age Related Healthcare, Adelaide and Meath Hospital, Tallaght, Dublin 24, Ireland; 3 School of Medicine, Trinity College Dublin, Dublin, Ireland

**Keywords:** Malaria, Imported illness, Older traveler, Delayed diagnosis

## Abstract

**Background:**

Increased co-morbidities and physiological changes mean older patients may be at higher risk of adverse outcomes from certain imported illnesses. One of the most commonly diagnosed imported infections in returning travelers is malaria. Increasing age is strongly and independently associated with increasing morbidity and mortality from malaria. Delayed diagnosis leads to higher risks of poor clinical outcomes in older patients presenting with malaria. The objective of this study was to quantify malaria presentations in older patients as a percentage of total malaria presentations, compare length of hospital stay (LOS) between the older and younger cohort, and to describe medical co-morbidities, length of time to diagnosis and factors contributing to delayed diagnosis and increased LOS in the older cohort.

**Methods:**

A retrospective cohort study was undertaken in two university hospitals of all patients aged 65 years or older presenting with malaria from 2002–2012. A national hospital inpatient database was used to identify patients of all ages with a discharge diagnosis of malaria over this ten year period, and quantify LOS in those aged <65 and those aged 65 years or older. The case-notes for all of the older cohort were reviewed.

**Results:**

There were a total of 203 cases, 12 of whom were aged ≥65 years (5.9 %- total). Median time to diagnosis in this older group was two days (range 0–35), median LOS was eight days (range 1–77), compared to a median LOS of three days in those aged <65 years. All patients aged ≥65 years presented with fever. Travel history was documented in only 6/12 charts, and 11/12 had two or more co-morbid illnesses. Six of the 12 patients were not diagnosed or treated within 48 h of presentation.

**Conclusions:**

This case series highlights the need for appropriate history-taking and timely diagnosis of the older traveler returning with fever, as delayed diagnosis and treatment can contribute to prolonged hospital stay and increased morbidity. With increasing numbers of older travelers, physicians must remain vigilant to the presence of imported illnesses, particularly malaria, in older patients with unexplained fever.

## Background

Older individuals account for a significant proportion of travelers [1–3] and the number of older people traveling is increasing, in keeping with increasing numbers of travelers of all age [4]. There is greater access to global malaria-endemic areas, and increased bi-directional migration between regions. Due to increased number of co-morbidities and physiological changes, these older patients are at higher risk of adverse outcomes from certain imported illnesses, particularly malaria [3], therefore it is of paramount importance to diagnose these infections as quickly as possible. One of the most commonly diagnosed imported infections in febrile travellers returning to Europe is malaria [4–7]. Malaria, in particular malaria due to *Plasmodium falciparum*, is associated with a high rate of morbidity and mortality. Increasing age is strongly and independently associated with increasing risk of death from malaria [8–11]. Prior studies have reported higher risks for severe malaria and adverse outcomes in older patients presenting with malaria [12–17], particularly in the setting of delayed diagnosis [9] and therefore any delay to diagnosis can be critical. Appropriate targeted history taking in any patient returning from abroad, and most importantly those presenting with fever, can lead to early diagnosis of imported illnesses, and thereby improve clinical outcomes, decrease length of hospital stay, and decrease overall morbidity and mortality.

The objective of this study was to quantify malaria presentations in older patients as a percentage of total malaria presentations, and to describe medical co-morbidities, length of time to diagnosis and length of hospital stay in the older cohort. Each case of malaria in an older patient was reviewed in detail to assess whether travel history was being considered in older patients, to quantify delays to diagnosis and treatment, and to assess the impact of patient demographic factors and medical co-morbidities, in order to identify reasons for poorer clinical outcomes.

## Methods

First, a Hospital In-patient Enquiry (HIPE) was used to identify all patients with a discharge diagnosis of malaria from 2002–2012 in two university hospitals in the Republic of Ireland. HIPE is a national database designed to collect demographic, clinical and administrative data on discharges from acute public-sector hospitals nationally in the Republic of Ireland [18]. Secondly, the case-notes of those aged ≥65 years who were initially identified by HIPE were reviewed. A case report form was designed to collect information about patient demographics and co-morbidities, presenting features, time to diagnosis, potential factors contributing to delayed diagnosis, and length of hospital stay (LOS) in the older population. The cases of malaria in patients aged <65 were quantified from the HIPE data for comparison with respect to length of stay.

The extraction of information from the case-notes was done by the main researcher, who was a medical doctor working in the two hospitals. The case report forms did not contain any personal identifying information, and therefore all datasets generated were anonymous.

Statistical analysis was performed using IBM SPSS statistics version 20®.

The study was in accordance with the ethical standards of the institutions and was deemed exempt from the need for further approval from the ethics review board.

## Results

There were a total of 203 cases of malaria over the examined ten year period, 12 of whom were aged 65 years or older (5.9 %).

### Patient characteristics in older cohort

Ten patients (83.3 %) were Caucasian, two (16.6 %) were Black; seven (58.3 %) were male. The median age was 67.5 (range 65–80). Twenty-five percent (3/12) were returning missionaries. All patients presented with a history of fever, and 10/12 patients had a documented fever on presentation. (Median temperature at presentation 38.4 degrees Celsius). Other common presenting features were malaise (10/12), headache (9/12), gastro-intestinal symptoms (8/12), and less commonly arthralgia (2/12) and jaundice (2/12). The patients had a median of 2.5 days of symptoms prior to presentation (range 1–7). Ninety-two percent (11/12) of the patients had two or more significant co-morbid illnesses, including hypertension (*n* = 5), respiratory disease (*n* = 4), ischaemic heart disease (*n* = 4), lipid disorders (*n* = 3), lymphoma (*n* = 1), haematological disorders (*n* = 2) and hepatitis C virus infection (*n* = 1).

### Time to diagnosis and clinical course in older cohort

Median time from presentation to diagnosis was two days (range 0–35) in the older cohort. Only 50 % (6/12) of patients were diagnosed on admission. Of those who were not diagnosed on admission, the median time to diagnosis was five days (range 2–35). Factors contributing to delayed diagnosis included coexistent confounding illness and lack of awareness of travel history. In half of the cases (6/12) a travel history was not documented. As above, almost all patients (92 %) had co-morbidities, and of the six who were not diagnosed on admission, five of these were commenced on treatment for alternative diagnoses, and one was admitted for further investigation. None of the patients who had a delay to diagnosis were commenced on empiric treatment for malaria, nor was malaria documented in the differential diagnosis. Two cases were later incidentally diagnosed when the laboratory noticed trophozoites of *Plasmodium falciparum* on full blood count. One of these was a complicated case of Hepatitis C seroconversion who presented with jaundice. Hepatitis C was diagnosed prior to, and may have delayed, the subsequent diagnosis of malaria. The patient had a prolonged stay with coagulation defects and severe hospital-acquired pneumonia, and an eventual finding of plasmodium falciparum trophozoites on blood film on day 35 of admission. The patient was finally discharged home well after a 77-day hospital admission. One patient who presented with a history of fever but otherwise quite well was initially sent home, and was only diagnosed on re-presentation with worsening symptoms.

With regards to the type of malaria, all patients had *Plasmodium falciparum* trophozoites on blood film, and one patient was co-infected with *Plasmodium vivax*. This patient had a previous history of *Plasmodium vivax* infection, which had been treated abroad four years earlier. It is unclear whether this presentation was relapse or re-infection. In the six cases where travel history was documented, two had documented use of anti-malarial prophylaxis, two had not used prophylaxis, and for the final two there was no documentation about use of prophylaxis.

### Length of stay and factors contributing to prolonged hospital stay

Median length of hospital stay in the ≥65 s was eight days (range 1–77), compared to a median LOS of three days (range 1–11) in the <65 yr cohort . Reasons for the prolonged stay included the development of associated infections (3/12 patients), delays in identifying the cause of fever and therefore delays to initiation of treatment (as above). Added to this was a slow recovery phase once malaria treatment was instituted, possibly due to decreased resilience and increased medical co-morbidities. Despite varying clinical courses and complications, all 12 patients were eventually discharged home well.

## Discussion

Our retrospective case review identified a small but significant proportion of cases of imported malaria occurring in patients aged ≥65 years, as have other studies [3, 4, 8–10, 13–16]. It is well-established that older patients have worse clinical outcomes when presenting with imported malaria. Checkley et al. looked at over 25,000 patients with Plasmodium falciparum malaria presenting in the United Kingdom (UK) between 1987 and 2006, and case fatality rate was shown to be 4.6 % in those aged >65 years [8]. According to the European Network on Imported Disease Surveillance Data, the risk in elderly patients is almost six times higher in the >60 age group [13]. Similarly Saliba et al. showed that age >40 years was independently predictive of severe malaria [17]. While none of our small cohort died, their collective LOS and complicated clinical courses support the existing data on worse morbidity in the older age group.

We found a high LOS in the patients aged ≥65 years, and significant delays in time from presentation to diagnosis. A literature review by Luthi et al. showed that age, delay in seeking care, delayed diagnosis, and delayed administration of the correct treatment are some of the main risk factors for fatal malaria in travellers [10]. Other studies looking at delays to diagnosis have mainly commented on delay between onset of symptoms and patient seeking care [9, 15]. A US study by Newman et al. showed a median of 4.5 days from symptom onset to presentation, which is higher than our median of 2.5 days [19]. A cohort study in France conducted between 1996 and 2003, which included more than 20,000 patients, found that the median time from onset of symptoms to diagnosis was three days, but this was not broken down into time to presentation and time from presentation to diagnosis [9]. The indicator that we are presenting (delay from presentation to diagnosis) could be considered a useful one in evaluating the performance of the secondary/tertiary health care system.

A small number of other studies have looked at delays to diagnosis and/or treatment after presentation to a health care facility. In a US study of malaria-related deaths among travelers from 1963–2001, a remarkable 67.8 % of persons who died did not obtain a correct diagnosis on the day of presentation (range 1–17 days to achieve the correct diagnosis) [19]. In a paper by Santos et al. the mean time between arrival and diagnosis was eight days in patients who survived, and 16 days in patients who had a fatal outcome ie) delayed diagnosis was associated with a fatal outcome [20]. With regard to reasons for this delay, in our study the main contributing factors were co-morbidities and confounding illness (11/12 patients had two or more co-morbidities, and five patients were initially diagnosed with pathologies other than malaria), and the lack of awareness of travel history (we could not establish for how many patients travel history was not collected as the absence of travel history in the chart could mean either no travel history or that the patient was not asked). In a Swiss study, 2/11 cases of malaria that had unusual transmission were fatal, and this high case fatality rate was thought to be related to a late diagnosis due to missing history or a history that did not point towards malaria as a possibly diagnosis [21]. The coexistence of a confounding illness is a challenge that clinicians face daily, not only in these specific presentations. Detailed history taking is vital for rapid diagnosis of any presenting illness, and, clearly, omitting a travel history means that vital diagnostic information is missing.

As expected, we found longer lengths of stay in this older population (median eight days versus median three days in the under 65 year age group). The median stay of three days in the younger cohort is in keeping with median LOS found in other studies, such as a French cohort study where the median length of stay across all age groups was three days [9]. To our knowledge no other studies have commented on LOS with specific reference to or comparison with older individuals. The aforementioned co-morbidities also very likely contributed to the longer LOS in the older cohort, along with host-specific factors in older patients leading to different immunological responses and levels of parasitaemia [22, 23].

In our study, the delay from first presentation to diagnosis correlated with a delay in treatment initiation, which also had an impact on the longer LOS in this group. A large UK-based observational study showed that for the most part, prompt treatment followed a diagnosis of severe malaria in a specialist centre (median diagnosis-to-treatment time was 1 h (range 0.5-5) for patients receiving artesunate in the specialist centre). Significant delays from diagnosis to treatment were only seen in regional centers who did not have artesunate on site. The fact that treatment promptly followed diagnosis in specialist centers is generalisable to our study, which was performed in two tertiary referral centers, and therefore we can assume that our delays in treatment were due to delay in diagnosis [24].

Our study did not calculate the median delay to diagnosis in the younger age-group, which did not allow for direct comparison. However, we do know that the median delay from presentation to diagnosis was two days in the older group, and the median LOS in the younger group was three days. Therefore if the patients with malaria from the younger cohort were discharged on day three (median) after presentation we can assume that their correct diagnosis must have been secured for the most part on day one.

This study has other limitations, not least of which are its small numbers. This, and the fact that the study was conducted in only two centers, means that caution must be taken with generalizing results. However, even in settings that would expect to see even fewer presentations of imported infection, it is important for physicians to remain alert to the possibility; a large study in the UK showed that while most UK cases are seen in hospitals which see a lot of malaria cases, 25 % of falciparum malaria is seen in centers which see less than one malaria patient a month, and 6 % in hospitals that see fewer than two cases per year [24]. Furthermore case-notes reviews have their limitation; the non-documentation of questions relating to travel history and use of malaria prophylaxis were taken to mean that these questions were not asked, which may not be the case. Finally the HIPE system also has limitations – use of this system only captures data pertaining to patients on whom a discharge summary was completed by medical staff.

## Conclusion

Older individuals represent a small but substantial proportion of travelers, and subsequently of patients presenting with imported infections such as malaria. As worldwide travel becomes more accessible older individuals are likely to represent an increasing proportion of patients returning with imported illness. As in any case, early diagnosis is essential, and is largely dependent on detailed accurate travel history retrieval. This case series reiterates the importance of early consideration of the possibility of malaria in at-risk patients as a delay to diagnosis can result in prolonged hospital stay and increased morbidity.

This case series also highlights certain challenges to the identification of imported infection in older patients, such as the varied presenting features, especially in patients with other co-morbid illnesses, as well as the possible omission of a quick but vital travel history. While this study suggests that older patients still only account for a small proportion of patients diagnosed with malaria in these centers, with increasing numbers of older travelers this proportion may increase in line with the general ageing population over the coming years. Physicians must remain vigilant to the potential presence of imported infections, especially malaria, in both younger and older patients with unexplained fever and a history of travel.

## Abbreviations

HIPE: hospital inpatient enquiry
LOS: length of hospital stay
UK: United Kingdom
US: United States of America
